# Proposal of Allobogoriella gen. nov., Allobogoriella caseilytica comb. nov., Allostella gen. nov., Allostella humosa comb. nov. and Allostella vacuolata comb. nov. as replacement names for the illegitimate prokaryotic names Bogoriella, Bogoriella caseilytica, Stella, Stella humosa and Stella vacuolata, respectively

**DOI:** 10.1099/ijsem.0.006930

**Published:** 2025-10-01

**Authors:** Umakant Bhoopati Deshmukh, Aharon Oren

**Affiliations:** 1 Institution of Higher Learning, Research and Specialized Studies Centre, Department of Botany, Janata Mahavidyalaya, Chandrapur 442 401, India; 2 Department of Plant and Environmental Sciences, The Institute of Life Sciences, The Hebrew University of Jerusalem, Edmund J. Safra Campus, Jerusalem 9190401, Israel

**Keywords:** *Bogoriella*, *Bogoriellaceae*, illegitimate names, *Stella*, *Stellaceae*

## Abstract

The prokaryotic generic names *Bogoriella* Groth *et al*. 1997 and *Stella* Vasilyeva 1985 are illegitimate because they are later homonyms of the genus names *Bogoriella* Zahlbr. 1928 (Ascomycota) and *Stella* Massee 1889 (Basidiomycota) (Principle 2 and Rule 51b(4) of the International Code of Nomenclature of Prokaryotes). We therefore propose the replacement names *Allobogoriella* gen. nov., *Allobogoriella caseilytica* comb. nov., *Allobogoriellaceae* fam. nov., *Allobogoriellales* ord. nov., *Allostella* gen. nov., *Allostella humosa* comb. nov. and *Allostellaceae* fam. nov. as replacement names for the illegitimate prokaryotic names *Bogoriella*, *Bogoriella caseilytica*, *Bogoriellaceae*, *Bogoriellales*, *Stella*, *Stella humosa*, *Stella vacuolata* and *Stellaceae*, respectively.

The prokaryotic genus *Bogoriella* (*Bogoriellaceae*, *Micrococcales*, *Actinomycetes*, *Actinomycetota*) was described by Groth *et al*. in 1997 [1]. The genus currently contains one species with a validly published name: *Bogoriella caseilytica*. The family *Bogoriellaceae* was established to accommodate this genus [2]. The genera *Georgenia* Altenburger *et al*. 2002 and *Oceanitalea* Fu *et al*. 2012 also belong to this family, which was classified in the order *Bogoriellales* [3].

The generic name *Bogoriella* is a homonym of *Bogoriella* Zalhbr. 1928 (Ascomycota) [4] (Mycobank # 608). Based on Principle 2 and Rule 51b(4) of the International Code of Nomenclature of Prokaryotes (ICNP) [5] and the version of the International Code of Nomenclature of Bacteria in effect at the time [6], the name *Bogoriella* proposed in 1997 for a prokaryotic genus is therefore illegitimate. The ICNP offers two possibilities to resolve the illegitimacy of a name: by replacing an illegitimate name with a new legitimate one or by addressing a request for an opinion to the Judicial Commission to obtain an exception allowing the illegitimate name to be conserved [7,8]. As the replacements proposed in this manuscript are not expected to cause errors or confusion, we prefer the first option here. In accordance with Rule 54, we here propose a replacement name, conserving the meaning of the illegitimate name as originally published. We also propose replacement names for the family *Bogoriellaceae* Schumann and Stackebrandt 2000 and the order *Bogoriellales* Salam *et al*. 2020 that have *Bogoriella* as the type genus.

## Description of *Allobogoriella* Deshmukh and Oren gen. nov.

*Allobogoriella* (Al.lo.bo.go.ri.el’la. Gr. masc. adj. *allos*, other; N.L. fem. n. *Bogoriella*, a bacterial genus, named after Lake Bogoria in Kenya; N.L. fem. n. *Allobogoriella*, another *Bogoriella*).

The description of the genus is as given for *Bogoriella* [1].

The type species is *Allobogoriella caseilytica* (Groth *et al*. 1979) Deshmukh and Oren.

## Description of *Allobogoriella caseilytica* (Groth *et al*. 1979) Deshmukh and Oren comb. nov.

*Allobogoriella caseilytica* [ca.se.i.ly’ti.ca. L. masc. n. *caseus*, cheese; N.L. masc. adj. *lyticus* (from Gr. masc. adj. *lytikos*), able to loosen, able to dissolve; N.L. fem. adj. *caseilytica*, loosening or dissolving casein].

Basonym: *Bogoriella caseilytica* Groth *et al*. 1979.

The description of the species is as given for *Bogoriella caseilytica* [1].

The type strain is HKI 0088^T^ (=ATCC 700413^T^=CIP 105404^T^=DSM 11294^T^=JCM 11479^T^), isolated from Lake Bogoria, Kenya.

The 16S rRNA gene sequence accession number of the type strain is Y09911.

The genome sequence of the type strain can be accessed from GenBank as RKHK00000000.

## Description of *Allobogoriellaceae* fam. nov.

*Allobogoriellaceae* (Al.lo.bo.go.ri.el.la.ce’ae. Gr. masc. adj. *allos*, other; N.L. fem. pl. n. *Bogoriellaceae*, a bacterial family name; N.L. fem. pl. n. *Allobogoriellaceae*, another *Bogoriellaceae* family).

The description of the family is as given for the family *Bogoriellaceae* [2].

The type genus is *Allobogoriella* Deshmukh and Oren.

## Description of *Allobogoriellales* ord. nov.

*Allobogoriellales* (Al.lo.bo.go.ri.el.la’les. Gr. masc. adj. *allos*, other; N.L. fem. pl. n. *Bogoriellales*, a bacterial order name; N.L. fem. pl. n. *Allobogoriellales*, another *Bogoriellales* order).

The description of the order is as given for the family *Bogoriellales* [3].

The type genus is *Allobogoriella* Deshmukh and Oren.

The prokaryotic genus *Stella* (*Stellaceae*, *Rhodospirillales*, *Alphaproteobacteria*, *Pseudomonadota*) was described by Vasilyeva in 1985 [9]. The genus currently contains two species with validly published names: *Stella humosa* and *Stella vacuolata*. To accommodate this genus and the genus *Constrictibacter* Yamada *et al*. 2011, the family *Stellaceae* was established [10,11].

The generic name *Stella* is a homonym of *Stella* Massee 1889 (Basidiomycota) [12] (Mycobank # 19327), a synonym of *Scleroderma* Pers. 1801 (Mycobank # 19309). Based on Principle 2 and Rule 51b(4) of the ICNP [5] and the version of the International Code of Nomenclature of Bacteria in effect at the time [13], the name *Stella* proposed in 1985 for a prokaryotic genus is therefore illegitimate. In accordance with Rule 54, we here propose a replacement name, conserving the meaning of the illegitimate name as originally published. We also propose a replacement name for the family *Stellaceae* Hördt *et al*. 2020 that has *Stella* as the type genus.

## Description of *Allostella* Deshmukh and Oren gen. nov.

*Allostella* (Al.lo.stel’la. Gr. masc. adj. *allos*, other; L. fem. n. *Stella* (‘star’), a bacterial genus; N.L. fem. n. *Allostella*, another *Stella*)

The description of the genus is as given for *Stella* [9].

The type species is *Allostella humosa* (Vasilyeva 1985) Deshmukh and Oren.

## Description of *Allostella humosa* (Vasilyeva 1985) Deshmukh and Oren comb. nov.

*Allostella humosa* (hu.mo’sa. L. fem. n. *humus*, soil, earth; L. masc. adj. suff. *-osus*, suffix used with the sense of full of, prone to; N.L. fem. adj. *humosa*, intended to mean pertaining to soil or earth).

Basonym: S*tella humosa* Vasilyeva 1985.

The description of the species is as given for *Stella humosa* [9].

The type strain is AUCM B-1137 (=ATCC 43930^T^=DSM 5900^T^=VKM B-1137^T^), isolated from soil.

The 16S rRNA gene sequence accession number of the type strain is AJ535710.

The genome sequence of the type strain can be accessed from GenBank as AP019700.1 [14].

## Description of *Allostella vacuolata* (Vasilyeva 1985) Deshmukh and Oren comb. nov.

Basonym: S*tella vacuolata* Vasilyeva 1985.

The description of the species is as given for *Stella vacuolata* [9].

The type strain is 229 (=ATCC 43931^T^=DSM 5901^T^=VKM B-1552^T^), isolated from a piggery.

The 16S rRNA gene sequence accession number of the type strain is AJ535711.

The genome sequence of the type strain can be accessed from GenBank as AP019702.1 [14].

## Description of *Allostellaceae* fam. nov.

*Allostellaceae* (Al.lo.stel.la.ce’ae. Gr. masc. adj. *allos*, other; N.L. fem. pl. n. *Stellaceae*, a bacterial family name; N.L. fem. pl. n. *Allostellaceae*, another *Stellaceae* family).

The description of the genus is as given for the family *Stellaceae* [10].

The type genus is *Allostella* Deshmukh and Oren.

## Abbreviations

ICNP: International Code of Nomenclature of Prokaryotes
